# Live strong and prosper: the importance of skeletal muscle strength for healthy ageing

**DOI:** 10.1007/s10522-015-9631-7

**Published:** 2016-01-20

**Authors:** Michael McLeod, Leigh Breen, D. Lee Hamilton, Andrew Philp

**Affiliations:** MRC-ARUK Centre for Musculoskeletal Ageing Research, University of Birmingham, Birmingham, B15 2TT UK; School of Sport, Exercise and Rehabilitation Sciences, University of Birmingham, Birmingham, B15 2TT UK; School of Sport, University of Stirling, Stirling, UK

**Keywords:** Skeletal muscle, Strength, Health, Protein synthesis, Physiology, Protein metabolism

## Abstract

Due to improved health care, diet and infrastructure in developed countries, since 1840 life expectancy has increased by approximately 2 years per decade. Accordingly, by 2050, a quarter of Europe’s population will be over 65 years, representing a 10 % rise in half a century. With this rapid rise comes an increased prevalence of diseases of ageing and associated healthcare expenditure. To address the health consequences of global ageing, research in model systems (worms, flies and mice) has indicated that reducing the rate of organ growth, via reductions in protein synthetic rates, has multi-organ health benefits that collectively lead to improvements in lifespan. In contrast, human pre-clinical, clinical and large cohort prospective studies demonstrate that ageing leads to anabolic (i.e. growth) impairments in skeletal muscle, which in turn leads to reductions in muscle mass and strength, factors directly associated with mortality rates in the elderly. As such, increasing muscle protein synthesis via exercise or protein-based nutrition maintains a strong, healthy muscle mass, which in turn leads to improved health, independence and functionality. The aim of this review is to critique current literature relating to the maintenance of muscle mass across lifespan and discuss whether maintaining or reducing protein synthesis is the most logical approach to support musculoskeletal function and by extension healthy human ageing.

## Introduction

The percentage of the global population above 65, 85 and 100 years is predicted to increase by 188, 551 and 1004 % respectively by 2050 (The United Nations; World Population Prospects: http://esa.un.org/unpd/wpp/). As a consequence, globally, there is a notable increase in the prevalence of ‘diseases of ageing’, such as sarcopenia, recently been defined by the European Working Group on Sarcopenia in Older People (EWGSOP) as a ‘syndrome characterised by progressive and generalised loss of skeletal muscle mass and strength with the risk of adverse outcomes such as physical disability, poor quality of life and death’ (Baumgartner et al. 1998; Cruz-Jentoft et al. 2010; Rosenberg 1989). This onset of sarcopenia is fundamentally important for health as skeletal muscle in a healthy adult accounts for approximately 40 % of total body mass (Janssen et al. 2000). In addition to its primary tasks of maintaining posture, breathing and locomotion, skeletal muscle also represents an important nutrient store and metabolic regulator (Wolfe 2006). During ageing, approximately 30 % of an individual’s peak muscle mass is lost by the age of 80, and this loss is exacerbated by physical inactivity and poor nutrition (Janssen et al. 2000; Topinkova 2008). This decline in skeletal muscle metabolism and function should not be underestimated, as in the UK alone, complications arising from falls in the elderly (with an association to frailty) are estimated to cost the National Health Service £1.7 billion annually (www.ageuk.org.uk).

## The effect of ageing on human health

Ageing is characterized by widespread reduction in the reserve capacity of the body’s major organs (Topinkova 2008). Of critical importance for lifespan is the reduction in cardiac output (Lambert and Evans 2005), which together with reduced lung function (Taylor and Johnson 2010), decreased skeletal muscle oxidative capacity (Betik and Hepple 2008), and changes in body composition (Kuk et al. 2009) ultimately lead to a reduction in maximum oxygen consumption (VO_2_max) (approximately 1 % decline per year post 25 years) (Lambert and Evans 2005). VO_2_max (or surrogate measures of VO_2_max) correlate highly with mortality risk (Lee et al. 1999; Lee et al. 2010; Lee et al. 2011). These metabolic changes lead to a redistribution of nutrients, causing inappropriate fat deposition, which has been linked with systemic age related insulin resistance (Wolfe 2006).

Musculoskeletal deterioration in old age has severe health consequences. Skeletal muscle and the extracellular matrix of skeletal tendon-bone are essential for maintaining tissue structure and vital for muscular contraction and force transmission. Given the close link between muscle loss (sarcopenia) and bone loss (osteopenia), factors that affect muscle anabolism are also likely to effect bone mass. With advancing age, sarcopenia and osteopenia present major clinical problems, such as impaired locomotory function, compromised balance, increased risk of osteoarthritis and fall/fractures; all of which diminish quality of life in seniors (Cruz-Jentoft et al. 2010; Janssen et al. 2002; Landi et al. 2012a, b; Panel on Prevention of Falls in Older Persons and British Geriatrics 2011).

**Fig. 1:**
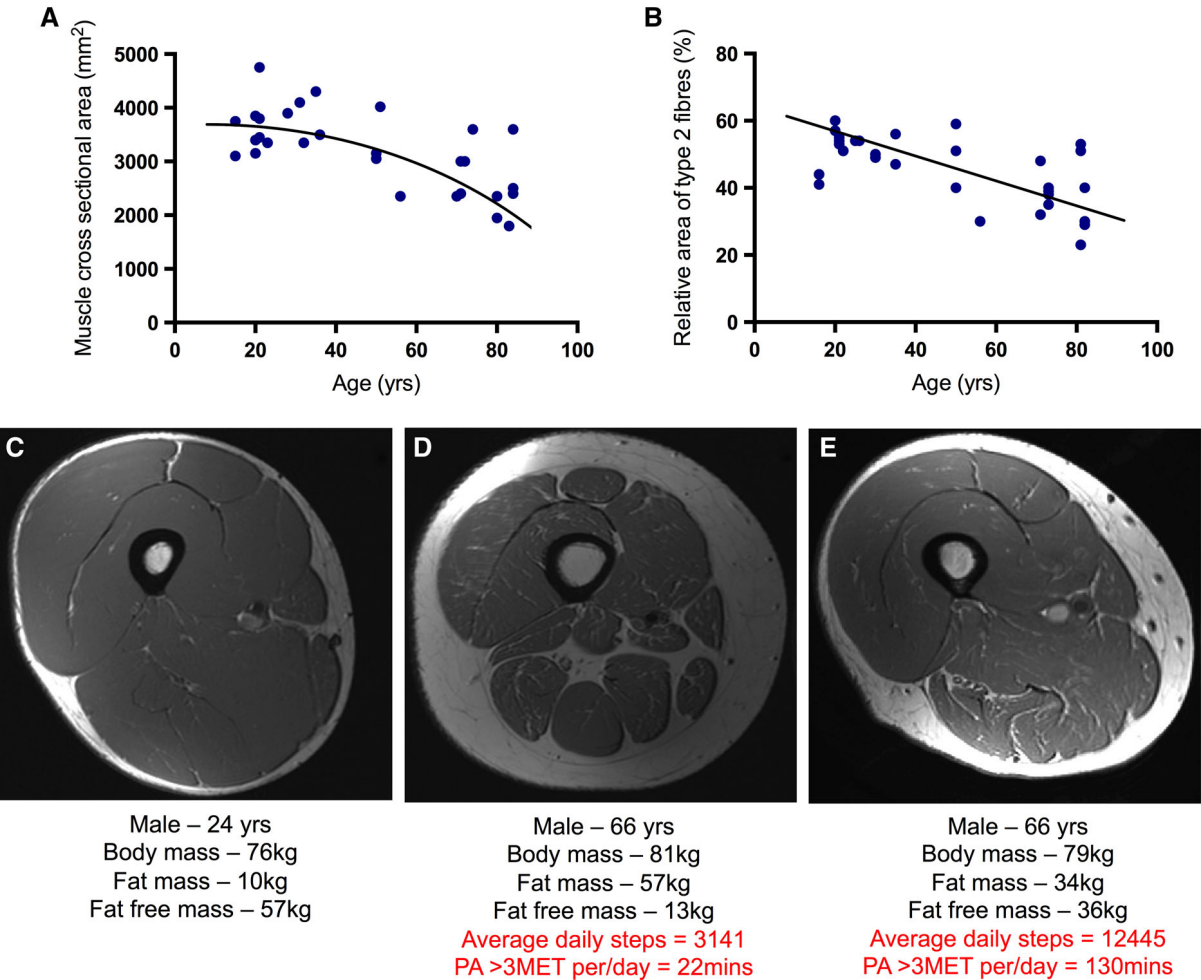
Loss of skeletal muscle size and quality occurs during healthy ageing. Skeletal muscle cross sectional area (CSA) declines across lifespan (**a**) with a preferential decline observed in type 2 fibres (**b**). Representative MRI images depict skeletal muscle architecture in young (**c**), old-inactive (**d**) and old-active (**e**) males. **a** and **b** are adapted from (Lexell 1995). Text represents subject characteristics relating to the images in **c**–**e**

Even in conditions of ‘healthy’ ageing, there is a progressive decline in skeletal muscle quality as described by various changes in structure, mechanics and function. Lexell (1995) observed that males aged 15–83 years displayed an age-related reduction in muscle cross sectional area progressing after 25 years (Fig. 1a). This was primarily caused by a loss in the number of fibres but also a reduction in relative cross-sectional area, particularly of type II fibres (Fig. 1b). The loss in fibre number and preferential loss in type II fibres may be related to changes in innervation, as with increasing age comes a loss of innervation of muscle fibres and a progressive loss of alpha-motorneurons (Brown 1972; Tomlinson and Irving 1977; Einsiedel and Luff 1992). Following the loss of alpha motorneurons, muscle fibres may become reinnervated by surrounding neurones in a cycle of denervation and reinnervation via collateral reinnervation (Holloszy and Larsson 1995) which likely contributes to the loss of strength and muscle mass with age (Luff 1998). With less motor neurones, the number of muscle fibres per motor unit increases, resulting in larger, less differentiated motor units (Andersen 2003). The preferential loss in type II fibre cross-sectional area may partly explain why age-associated losses in muscle strength and power occur at a greater and disproportionate rate to losses in muscle mass (Macaluso and De Vito 2004) and why aged muscle tends to show exacerbated fatigue resistance (Avin and Law 2011). As well as the above, numerous other factors including a reduction in number of satellite cells (Kadi et al. 2004), a potential shift towards slow myosin isoforms (Gelfi et al. 2006) and shortening of sarcomere length (Narici et al. 2003) have all be suggested to contribute to a reduction in force generating capacity of muscle tissue with ageing. It is extremely concerning that due to a loss of muscle strength with age, 16–18 % of women and 8–10 % of men over aged 65 cannot lift a 10 lb weight or kneel down (FIFoA-R 2008). This loss of muscle strength with ageing is known as dynapenia (Clark and Manini 2008), occurring 2–5 times faster rate than losses of muscle mass (Clark et al. 2006; Delmonico et al. 2009). The Health, Ageing and Body Composition study found that even gaining muscle mass with ageing does not entirely prevent ageing-related decreases in muscle strength (Delmonico et al. 2009). Infiltration of fat, and neural alterations are likely contributing factors, as well as changes in contractile properties (Kent-Braun et al. 2000), and many other mechanisms discussed elsewhere (Clark and Manini 2012; Mitchell et al. 2012). Dynapenia is a major risk factor for loss of dependence and mobility issues (Manini et al. 2007; Visser et al. 2005) as well as mortality (Newman et al. 2006; Takata et al. 2012).

Coupled to the loss of force generating capacity, there is a clear reduction of total muscle mass, at a rate of ~4.7 % peak mass/decade in men and ~3.7 % peak mass/decade in women (Mitchell et al. 2012). Graphical representation of this change in muscle size and composition with age is illustrated in Fig. 1c–e (Breen et al. *unpublished data*). Image 1c shows an MRI scan from a young lean male, in contrast to an age-inactive (1D) and age-matched active individual (1E), with similar levels of dietary protein intake [~0.9 g/(kg/body mass)]. Evident is the reduction in muscle mass with age (1C vs. 1D), the greater abundance and infiltration of fat around the muscle tissue (1C vs. 1D) and also the protective effect that maintained physical activity appears to have on skeletal muscle with ageing (1D vs. 1E). The accumulation of intramuscular fat may also be a key factor in the progressive mismatch between losses in mass and strength. Adipose tissue typically accumulates with age, producing numerous pro-inflammatory cytokines (adipokines) into the circulation which may accelerate muscle catabolism and contribute to a vicious cycle of muscle loss and fat accumulation (Schrager et al. 2007; Wellen and Hotamisligil 2003). Macrophage infiltration into the muscle as a result of increased lipid accumulation/adipokine has been termed ‘sarcopenic obesity’ (Baumgartner 2000; Stenholm et al. 2008). The combination of lipotoxocity and inactivity/ageing has been proposed to reduce skeletal muscle anabolic responses to resistance exercise and nutrition (Murton et al. 2015; Nilsson et al. 2013; Sitnick et al. 2009; Stephens et al. 2015). Figure 1e illustrates the preservation of muscle mass and reduced fat infiltration from a 66-year-old male age matched to Fig. 1d. The only major difference found between individuals from 1D and 1E is the habitual physical activity levels, with 1E ~4 times more active than 1D. Therefore, physical activity levels (coupled with good dietary practice) can maintain muscle mass and also strength in old age.

## The importance of skeletal muscle strength for healthy ageing

**Fig. 2:**
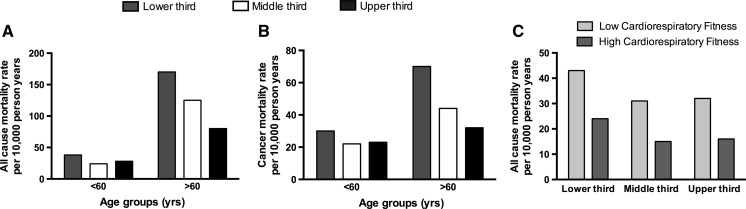
Skeletal muscle strength and cardiorespiratory fitness is associated with healthy ageing. Over the age of 60 years, all cause (**a**) and cancer-associated (**b**) mortality is twice as likely in individuals with low compared to high skeletal muscle strength. In addition, irrespective of strength, low cardio-respiratory fitness is associated with ~ twice the incidence of all cause mortality (**c**). Adapted from (Ruiz et al. 2008)

Ruiz et al. (2008) and coworkers carried out the most comprehensive study of its kind with over 8000 participants followed for approximately 18 years to assess the influence of muscle strength and cardiorespiratory fitness on healthy ageing (Ruiz et al. 2008). Subjects underwent a rigorous set of strength tests and were stratified by strength (Fig. 2a–b). Remarkably, individuals over the age of 60 years, classified in the lowest third for strength, were 50 % more likely to die of all cause mortality (Fig. 2a) than individuals in the upper third for strength (Ruiz et al. 2008). The same trend also applied when considering deaths associated with cancer (Fig. 2b), indicating that muscle strength, albeit correlative, has a protective effect from the incidence of cancer. A final key observation of this dataset was that regardless of strength, individuals with higher cardio-respiratory fitness had a greater life expectancy than low cardio-respiratory fitness counterparts (Fig. 2c). Collectively, this landmark study provided the first direct evidence that physical strength or the processes of developing strength is intrinsically linked to healthy ageing.

Muscle strength and VO_2_max are likely great predictors for life span because they integrate both the neuromuscular and cardiovascular systems and as such indicate the health and functional integration of these tissues. As we have discussed, one of the key determinants of health span is muscle strength (and likely muscle mass), and one of the major key determinants of muscle mass retention is the ability to modify muscle protein synthesis in response to anabolic stimuli. Given the clear benefits of muscle strength and cardiovascular fitness on healthy ageing, the obvious question is what approaches can be taken to preserve muscle mass, strength and function across lifespan?

### How is muscle mass, strength and function regulated at the molecular level?

The mechanistic target of rapamycin (mTOR) is a highly conserved serine/threonine kinase protein complex, identified as a central regulator of cellular growth (Fingar and Blenis 2004). Importantly, mTOR exists as 1 of 2 complexes and hyper-activity of the mTOR complexes 1 and 2 (mTORC1/2) has been implicated in tumor progression, pathological hypertrophy, diabetes and obesity (Lee et al. 2007; Sharp and Richardson 2011; Zoncu et al. 2011). mTORC1 and mTORC2 are very similar protein complexes differing in a few key subunits which alters kinase substrate preference. mTORC1 is the kinase component of both complexes and is a member of the phosphatidylinositol kinase (PIK) related kinases (Abraham 1996), although it does not possess lipid kinase activity (Brunn et al. 1997). The activity and substrate preference of mTOR is dependent upon several adapter proteins GβL (Kim et al. 2003), raptor (Hara et al. 2002), rictor (Sarbassov et al. 2004), Sin1 (Yang et al. 2006) and Protor/PRR5 (Pearce et al. 2007; Woo et al. 2007), which form two separate mTOR complexes capable of regulating distinctive pathways. The mTOR complex 1 contains GβL, raptor and mTOR and is rapamycin sensitive. GβL functions to stabilize the association between mTORC1 and raptor and enhances the kinase activity of mTORC1 towards its targets (Guertin et al. 2006), however is not essential for mTORC1 activity (Guertin et al. 2006). Raptor is an adapter protein that identifies and binds substrates that contain TOS (TOR signalling) motifs (Schalm et al. 2003) such as 4EBP and S6K1 (Schalm and Blenis 2002). mTORC2 on the other hand consists of mTOR, rictor, GβL, Sin1 and Protor/PRR5 and is rapamycin insensitive (Sarbassov et al. 2004).

mTORC1 regulates protein synthesis initiation by controlling the formation of the eIF4F complex (Gingras et al. 2004) and governs the pioneer round of mRNA translation by acting on SKAR via its putative target S6K1 (Ma et al. 2008). In addition, mTORC1 controls ribosomal biogenesis by regulating rDNA transcription in a manner dependant upon UBF (Hannan et al. 2003), and nuclear RNA export by regulating eIF4E in a manner dependant upon 4EBP1 phosphorylation (Culjkovic et al. 2005; Topisirovic et al. 2003; Topisirovic et al. 2004). In these roles mTORC1 is a critical regulator of protein synthesis and cell size (Fingar et al. 2002). It is well established that resistance exercise and provision of dietary protein activate mTORC1 synergistically, with the post exercise/post prandial activation of mTORC1 associated with increases in net protein synthesis in skeletal muscle (Brook et al. 2015).

### The importance of exercise in the maintenance of muscle mass and function

There is increasing evidence that the trajectory of sarcopenia and muscle loss is highly dependent on physical activity levels (Kortebein et al. 2008). Chronic sedentary behaviour and physical inactivity are key mechanistic drivers of sarcopenia, and can accelerate loss of muscle mass and strength leading to impaired mobility, higher risk of falls and increased mortality (Montero-Fernandez and Serra-Rexach 2013). Even acute bouts of inactivity such as 10 days of bed rest in older adults can substantially reduce lower leg strength, reduce aerobic capacity by 12 % and lead to a 7 % reduction of physical activity after the bed rest programme (Kortebein et al. 2008). Pharmacological interventions aimed to slow the progression of, or reverse sarcopenia, have been generally unsuccessful (Borst 2004; Onder et al. 2009). Clear and effective lifestyle-based counter-measures are therefore needed.

Repeated resistance exercise results in muscle protein accretion (i.e. hypertrophy) through chronically elevated rates of synthesis that exceed that rate of breakdown (Brook et al. 2015; Wilkinson et al. 2014). However, the sarcopenic elderly demonstrate an age-related anabolic resistance to exercise and protein ingestion (Cuthbertson et al. 2005; Kumar et al. 2009). Despite this anabolic resistance, the cumulative effects of chronic training with appropriate protein intake can promote muscle repair, muscle preservation and muscle growth in the elderly if a sufficient stimulus is maintained (Walker et al. 2011). In support of this, numerous studies have shown the beneficial effects of exercise in elderly individuals (Hakkinen et al. 1998) and even those aged >90 years (Fiatarone et al. 1994). Benefits include increased satellite cell content (Leenders et al. 2013) increased muscle cross-sectional area and myofiber differentiation (Kosek et al. 2006), increased muscle fibre size particularly in type II muscle fibers (Leenders et al. 2013) and greater muscle size and strength (Candow et al. 2006; Geirsdottir et al. 2012a). These are accompanied by metabolic adaptations such as increased metabolic rate (Hakkinen et al. 1998) mitochondrial biogenesis and efficiency of substrate metabolism (Holloszy and Coyle 1984???), and exercise capacity (McCartney et al. 1995). Long-term resistance training increases fibre dimension (Narici et al. 1996) strength and muscle function (Macaluso and De Vito 2004). Importantly, resistance exercise is also safe for use within the healthy elderly and possibly even in those with adverse cardiovascular signs or complications (Williams et al. 2007). In addition, resistance training has been observed to effectively improve balance (Orr et al. 2008) and in turn reduced fear of and occurrence of falling (Rubenstein et al. 2000), improve cognitive function (Cassilhas et al. 2007), decrease sit to stand time (Leenders et al. 2013), reduce risk of repeat and single hospitalisations (Lang et al. 2010) increase gait speed (Studenski et al. 2011) and most importantly improve overall quality of life (Geirsdottir et al. 2012b; Levinger et al. 2009). In addition, there is also growing evidence that the use of regular aerobic exercise can preserve muscle mass and function with age (Harber et al. 2009) a point we discuss in more detail in our recent review (Brook et al. 2015).

### The importance of dietary protein in the maintenance of muscle mass and function

Dietary protein is an essential macronutrient for the maintenance of muscle mass and function and, by close association, bone strength and density. The current recommended dietary allowance (RDA) for protein intake to meet whole-body metabolic demands is 0.8 g/(kg/day) (0.32/kg/LBM/day if assuming LBM accounts for 40 % of total body mass Janssen et al. 2000). However, the RDA does not distinguish between potential differences in the amount of protein required to maintain musculoskeletal health between young and older individuals. Given strong evidence that protein intakes greater than the current RDA are associated with multiple improved musculoskeletal health outcomes (Morley et al. 2010), it has been postulated that protein intakes should be defined in amounts that are ‘optimal’ to promote muscle/bone protein accretion, or at the very least, maintenance in old age.

Maintenance of musculoskeletal mass is dependent on nutrient-induced stimulation of muscle protein synthesis and the concomitant suppression of muscle protein breakdown to promote net protein accretion, which counters protein loss in the postabsorptive state. Protein-based nutrition robustly stimulates MPS through constituent essential amino acids (Chesley et al. 1992; Tipton et al. 1999). In particular, the branched-chain amino acid, leucine, displays potent muscle anabolic properties, and is capable of stimulating MPS and associated mTOR-mediated signaling in the absence of, and to a greater extent than, the other essential amino acids (Atherton et al. 2010; Wilkinson et al. 2013). The anabolic response of muscle to amino acid provision is relatively short lived, peaking at ~2 h after protein ingestion, returning to post-absorptive values by ~3 h after protein ingestion (Burd et al. 2010; Mitchell et al. 2015), even in situations where circulating amino acids remain elevated (Bohé et al. 2001).

A number of studies demonstrate that the muscle protein synthetic response to oral protein ingestion or essential amino acid infusion (to bypass potential age-related differences in splanchnic extraction) is markedly lower in the old compared with the young (Cuthbertson et al. 2005; Guillet et al. 2004; Katsanos et al. 2005; Volpi et al. 2000). However, it is important to note that not all studies have been able to detect the presence of age-related muscle anabolic resistance to protein-based nutrition (Paddon-Jones et al. 2004; Pennings et al. 2011), perhaps due to differences in the methods used for assessment of MPS between studies (Burd et al. 2012). Mechanisms beyond the intramuscular level may also underpin the compromised muscle anabolic response to protein nutrition in older individuals. In this regard, it has been demonstrated that ageing is associated with greater splanchnic extraction of amino acids, thereby reducing the appearance of dietary protein (Volpi et al. 1999). Beyond this, others report that microvascular perfusion following nutrient provision is impaired in older individuals (Mitchell et al. 2013), which in theory may limit the capacity for EAA delivery and uptake into muscle.

**Fig. 3:**
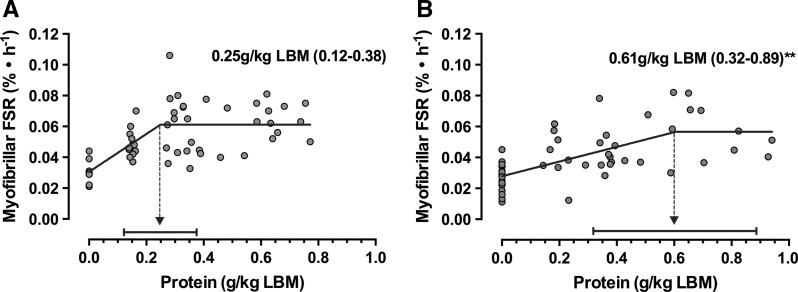
Ageing is associated with a blunted anabolic response to protein ingestion in humans. Comparative analysis indicates that the protein dose required to maximally stimulate myofibrillar fractional protein synthesis rates in young individuals (**a**) is 40 % lower than in old individuals (**b**). Data adapted from (Moore et al. 2015)

A recently published study shed important light on the debate of whether ‘anabolic resistance’ resides in old age, Moore et al. (2015) and colleagues performed a retrospective analysis of studies from a large cohort of young and old individuals in which MPS was measured following ingestion of varying amounts of high quality protein. The authors demonstrated that the minimal amount of protein required to reach a maximal MPS response was 0.25 g/kg/lean body mass (LBM) in young individuals and 0.61 g/kg/LBM in the old, thus highlighting the older individuals are less sensitive to low protein intakes, and therefore have a greater relative protein requirement than the young (Fig. 3). These data are all the more concerning when one considers that many older individuals do not meet the current RDA for protein due to factors including anorexia, appetite loss, blunted olfactory perception, gastrointestinal issues and, in some cases, socio-economic factors. On top of these concerning issues, many older individuals consume dietary protein in a skewed pattern (Tieland et al. 2012). For example, protein intake at breakfast and lunch is often sub-optimal to maximally stimulate MPS (<0.61 g/kg/LBM), a response that may only be achieved when large-dose protein is consumed with dinner (Tieland et al. 2012). Collectively, these data have led to suggestions that protein intake should perhaps be distributed evenly across each meal.

The presence of muscle anabolic resistance in old age has led to calls for the protein RDA to be increased in this population in order to maximally stimulate MPS and, perhaps, alleviate the progression of sarcopenia. Considering the data presented by Moore et al. (2015), a 70 year old individual weighing 80 kg (assuming LBM accounts for 30 % of total body mass (i.e. 24 kg) and three square meals per day (Short and Nair 2000)) would need to consume ~147 g of protein per day or ~1.8 g/kg, more than twice the current RDA. In support of this hypothetical scenario, Kim et al. (2015) recently demonstrated that consuming 1.5 g/(kg/body mass) of protein [almost double the current RDA—0.8 g/(kg/body mass)] resulted in markedly greater stimulation of MPS over 24 h compared with 0.8 g/(kg/body mass) of protein. In this study, no added muscle anabolic benefit was reported when protein was ingested evenly as compared with skewed distribution (Kim et al. 2015). Beyond acute studies of MPS, long-term investigations also lend weight to the suggestion that the current RDA for protein is inadequate to meet the metabolic needs of older individuals. For example, Campbell et al. (2001) demonstrated that older individuals consuming the recommended protein RDA for 14 weeks experienced a significant loss in mid-thigh muscle area. In addition, data from the Health ABC study highlighted that older individuals in the highest quintile for protein intake (~19 % of total energy intake) lost ~40 % less lean mass than did those in the lowest quintile for protein intake (~11 % of total energy intake) (Anderson et al. 2011).

It is clear that many older individuals do not consume sufficient protein to maintain whole-body and tissue-specific metabolic health, a situation that is worsened during periods of ill health and disability (Covinsky et al. 1999). Malnourishment during acute and chronic disease slows the rate of recovery, increases the risk of complications and re-admittance to hospital (Covinsky et al. 1999). Musculoskeletal unloading during illness and hospitalization occurs with greater frequency in older individuals and has dire consequences for musculoskeletal health. For example, it has been demonstrated that 7 days of bed rest induces muscle atrophy and anabolic resistance in older individuals (Drummond et al. 2012). Furthermore, we observed a similar loss in muscle mass and anabolic sensitivity in older individuals following 14 days of reduced ambulation (Breen et al. 2013). In addition to skeletal muscle defects, disuse events are also accompanied by severe osteopenia. Thus, deterioration in muscle anabolic responsiveness and bone properties during consecutive disuse events in old age may accumulate and drive the progression of sarcopenia and osteopenia. A general consensus gathering momentum is that high protein intakes (greater than the current RDA) are required during illness and hospitalization to maintain musculoskeletal health and quicken the rate of recovery (Cawood et al. 2012). This point is reinforced by evidence that muscle wasting is evident during disuse even with protein intakes of 1.0–1.2 g/(kg/body mass) (Trappe et al. 2007). From a clinical perspective, one study also reported that the sickest older hospital patients are the most protein malnourished (Pichard et al. 2004). Beyond skeletal muscle health, in osteoporosis there is evidence that higher bone mineral density is evident when protein intake exceeds the current RDA (Devine et al. 2005; Meng et al. 2009). Further, in hip fracture patients, supplemental or higher protein intakes (greater than the current RDA) are associated with increased bone density and a reduction in recovery time (Schurch et al. 1998). The notion that higher dietary protein intakes (particularly those high in sulphur-containing amino acids) may lead to blood acidification, calcium resorption from bones and osteoporosis (the so called ‘acid-ash hypothesis) has been roundly disregarded in several meta-analyses (Fenton et al. 2008; Fenton et al. 2010; Fenton et al. 2009a; Fenton et al. 2009b). Specifically, it is clear that the available evidence does not support a causal association between dietary acid load and osteoporotic bone disease. To summarize, there is a clear need for dietary protein intake above the current RDA in older individuals to maintain musculoskeletal health and quality of life, with experts recommending between 1.0 and 1.2 g/(kg body mass) for healthy older individuals (Bauer et al. 2013). Moreover, this need is likely to be increased further during periods where musculoskeletal mass is compromised, such as illness and hospitalization, with experts recommending between 1.2 and 2.0 g/(kg/body mass) in such situations (dependent on the severity of illness and extent of malnutrition) (Bauer et al. 2013).

### Should research focus be on improving lifespan or healthspan?

Based on the data discussed above, it seems clear that skeletal muscle mass and strength is vital for healthy ageing, and that exercise and dietary protein are key mediators of mTORC1-accociated increases in skeletal muscle protein synthesis. However, there is a large body of evidence in model organisms suggesting that strategies that blunt protein synthesis (mTORC1 inhibition, calorie restriction) increase lifespan and as such show promise as longevity promoting therapies (Kapahi et al. 2010).

For example, the mTORC1 inhibitor Rapamycin increases lifespan in a variety of models including yeast (Kaeberlein et al. 2005), *c. elegans* (Jia et al. 2004) and *Drosophila Melanogaster* (Kapahi et al. 2004). In mice, Rapamycin increases lifespan at 90 % mortality by 14 % for males and 9 % for females respectively, with survival increasing regardless of late-life (600 days of age) or mid-life (270 days of age) administration (Harrison et al. 2009). Consistent with the notion that reducing protein synthesis can increase lifespan, individually knocking out eIF4G and S6K1 (Selman et al. 2009) improves longevity in model organisms whilst individuals with mutations leading to low insulin like growth factor1 (IGF1) and insulin levels, key drivers of mTORC1 activity and protein synthesis during development, have reduced rates of cancer and diabetes (Guevara-Aguirre et al. 2011; Shevah and Laron 2007). Additionally, the offspring of centenarians (who have a delayed risk of developing certain diseases and an increased longevity against age matched controls) have low circulating IGF1 bioactivity (Vitale et al. 2012).

**Fig. 4:**
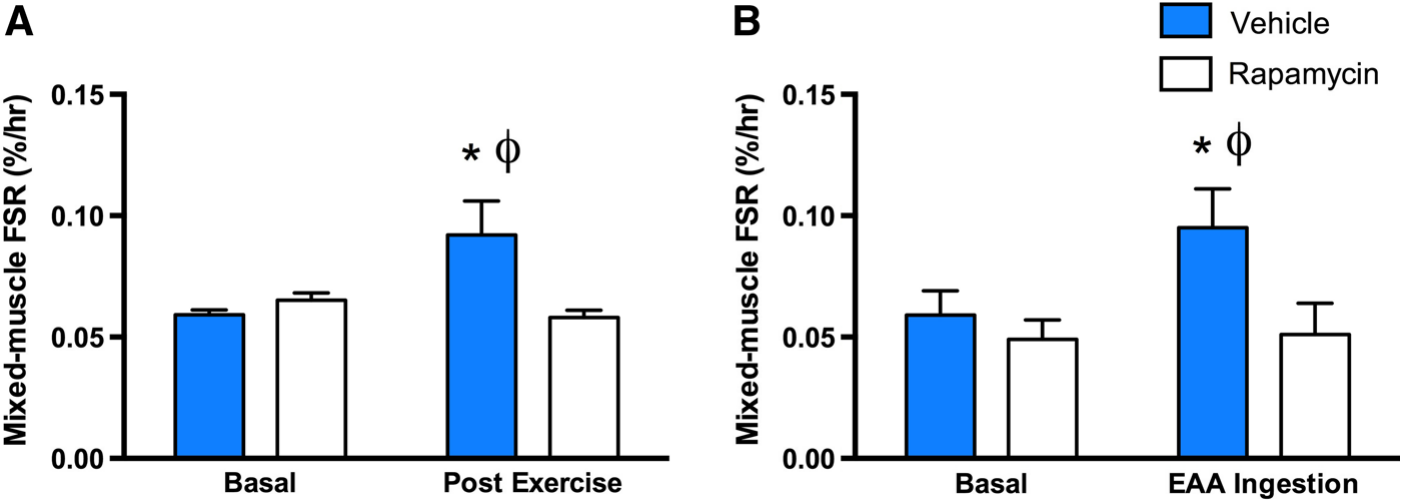
Inhibition of mTORC1 activity using the compound Rapamycin causes anabolic resistance in young, healthy males. Increases in mixed-muscle synthesis rates are blocked following resistance exercise (**a**) and essential amino acid (EAA) ingestion (**b**) in young healthy males. **a** Adapted from (Drummond et al. 2009); **b** Adapted from (Dickinson et al. 2011)

Some of the nuances in these various models/studies have recently been expertly reviewed elsewhere (Sharples et al. 2015), and what is clearly apparent is that few studies have taken into account muscle strength, size and functional capacity when considering improvements in lifespan. As such, the physiological context (i.e. healthspan vs lifespan) of living longer in these models is often overlooked. Two key studies from Blake Rasmussen’s lab; Dickinson et al. (2011) and Drummond et al. (2009) have experimentally tested the direct effect of rapamycin in human skeletal muscle (Fig. 4). First, Drummond et al. (2009), demonstrated that pretreatment of human subjects with rapamycin completely blocked the ability of resistance exercise to increase muscle protein synthesis (Fig. 4a) confirming previous reports in rat skeletal muscle (Kubica et al. 2005). Second, Dickinson et al. (2011), demonstrates that rapamycin could completely block the muscle protein synthetic response to essential amino acids (Fig. 4b). Whilst no studies have examined the long-term effects of rapamycin treatment on skeletal muscle in humans, mice on chronic rapamycin supplementation display reduced myofibrillar protein synthesis (Drake et al. 2013) and loose the ability to accrue muscle mass following synergist ablation (Bodine et al. 2001). So effectively, rapamycin is inducing anabolic resistance in young, healthy individuals. Therefore rapamycin treatment in old individuals, already displaying anabolic resistance and frailty appears to be a questionable therapeutic approach to improve healthspan.

With regard to calorie restriction, it also seems clear that the severity of calorie restriction employed and the macronutrient content administered has a dramatic effect on the preservation or loss of muscle mass (Cerqueira and Kowaltowski 2010). For example, there is some evidence that supplementing protein during periods of negative energy balance can preserve muscle mass in healthy human volunteers, compared to energy restriction alone (Josse et al. 2011; Mettler et al. 2010; Phillips 2014). Therefore, it would appear that future research into the macronutrient content of calorie restricted diets, in combination with greater understanding of skeletal muscle function is an important future direction for calorie restriction research.

A final point of consideration is that longevity studies in rodents are conducted in thermo-neutral, pathogen free environments, in conditions where food and water are abundant. Rodents are not required to forage for food and compete with littermates for survival (both processes that require functional skeletal muscle). In fact a recent report suggests that mice housed in such conditions have poor health and functional capacity (Martin et al. 2010). In such situations, loss of muscle mass and physiological function is not a detrimental factor. Collectively, this therefore raises the important question as to what we are striving for in this area of biogerontology? Are we looking to increase lifespan at a compromise of health-span, or would we rather live a healthy active life that encompasses maintenance of muscle mass, strength and function?

### Live strong and prosper

If we return to the data from Ruiz et al. (2008), this would suggest that maintaining physical strength is a key strategy that leads to healthy ageing. Couple this with the numerous pre-clinical and clinical human studies we have discussed, and it seems clear that an active lifestyle supported by appropriate dietary protein is the key to maintaining strong, healthy skeletal muscle. In this context, the importance of muscle size and strength for longevity and health in humans puts a new spin on the Darwinian statement “Survival of the Fittest” as it is clear that the strongest, fittest individuals are more likely to live longer and healthier lives (Artero et al. 2011; Artero et al.; Haskell et al. 2007; Ruiz et al. 2009; Ruiz et al. 2008). Given the highlighted importance of muscle function for healthspan, it is hoped that all future age-related research will consider skeletal muscle during experimental interventions and strive to use readouts of function as the principal outputs as opposed to isolated gene or protein analysis. Finally, and perhaps most importantly, when it comes to fully understanding the complexities of human ageing, it is clear that the only way to really achieve clarity is through integrative studies, from basic model systems through to complimentary detailed clinical studies in humans.
